# Beyond the clinic: a community-embedded, multidomain framework for early detection of glaucoma

**DOI:** 10.3389/fpubh.2026.1915134

**Published:** 2026-08-31

**Authors:** Akiko Hanyuda, Tetsuya Yamada, Satoru Tsuda, Noriko Himori, Toru Nakazawa

**Affiliations:** 1 Department of Ophthalmology, Keio University School of Medicine, Tokyo, Japan; 2 Department of Ophthalmology, Tohoku University Graduate School of Medicine, Sendai, Miyagi, Japan; 3 Head Office of Enterprise Partnerships, Tohoku University, Sendai, Miyagi, Japan; 4 Division of Ophthalmic Precision Medicine Development, United Centers for Advanced Research and Translational Medicine (ART), Tohoku University Graduate School of Medicine, Sendai, Miyagi, Japan; 5 Department of Aging Vision Healthcare, Tohoku University Graduate School of Biomedical Engineering, Sendai, Miyagi, Japan; 6 Department of Retinal Disease Control, Tohoku University Graduate School of Medicine, Sendai, Miyagi, Japan; 7 Department of Advanced Ophthalmic Medicine, Tohoku University Graduate School of Medicine, Sendai, Miyagi, Japan

**Keywords:** community health, fundus photography, genomics, glaucoma, living lab, normal-tension glaucoma, ocular blood flow, oxidative stress

## Abstract

Glaucoma remains one of the leading causes of acquired irreversible blindness worldwide, with normal-tension glaucoma representing the dominant subtype in Japan and several East Asian populations. The insidious, asymptomatic progression of this condition, combined with the demonstrated inadequacy of intraocular pressure alone as a screening criterion, creates a critical gap between disease burden and case detection. Population-based epidemiological studies consistently reveal that the majority of individuals with glaucoma are undiagnosed. Two responses have been suggested: incorporation of retinal imaging into annual health checkups, which warrants formal prospective evaluation, and characterization of individuals at higher risk — integrating genomic risk, oxidative stress biomarkers, systemic lifestyle factors, and ocular blood flow dynamics — which may help identify those in whom damage is most likely to occur. The principal contribution of this Perspective is therefore the implementation model rather than the individual screening components. We introduce the *Living Lab* (‘neighborhood health lab’), a community co-creation platform established under the Japan Science and Technology Agency COI-NEXT ‘Vision to Connect’ hub at Tohoku University, as a scalable model for operationalizing this framework. Embedded within commercial retail environments, the *Living Lab* integrates non-invasive screening, longitudinal health data collection, and evidence-based health product development—exemplified by the *Ronbun Recipe*® concept—within a stakeholder-aligned ecosystem encompassing citizens, researchers, industry, and municipal authorities. Conceived as a platform for well-being rather than as a disease-specific screening service, it engages individuals who are asymptomatic, undiagnosed, and outside existing screening pathways, and who would not otherwise be assessed at all.

## 1 Introduction

Glaucoma is a chronic, progressive optic neuropathy representing the second leading cause of blindness globally, affecting an estimated 80 million individuals, with projections exceeding 111 million by 2040 (1). Structural damage to the optic nerve and corresponding visual field loss are irreversible, rendering early detection the only viable strategy for preventing vision-threatening progression (2).

A defining epidemiological feature of glaucoma is its profoundly asymptomatic early course. Patients rarely perceive visual impairment until substantial nerve fiber layer loss has already occurred. Population-based prevalence studies, including landmark investigations from Japan such as the Tajimi Study and the Hisayama Study, have consistently demonstrated that more than 80% of individuals with glaucoma in the general population are unaware of their diagnosis (3, 4). This diagnostic gap persists despite the availability of effective treatments capable of halting progression.

Compounding this challenge is the epidemiological profile of glaucoma in East Asia. In contrast to Western populations, where high-tension glaucoma predominates, normal-tension glaucoma (NTG), defined by characteristic glaucomatous optic neuropathy and visual field loss in the absence of elevated intraocular pressure (IOP), accounts for most primary open-angle glaucoma (POAG) cases in Japan (3, 4). This distinction has profound implications for screening: conventional mass screening strategies that rely on IOP measurement as a proxy marker will inherently fail to detect the affected Japanese individuals.

**Figure 1:**
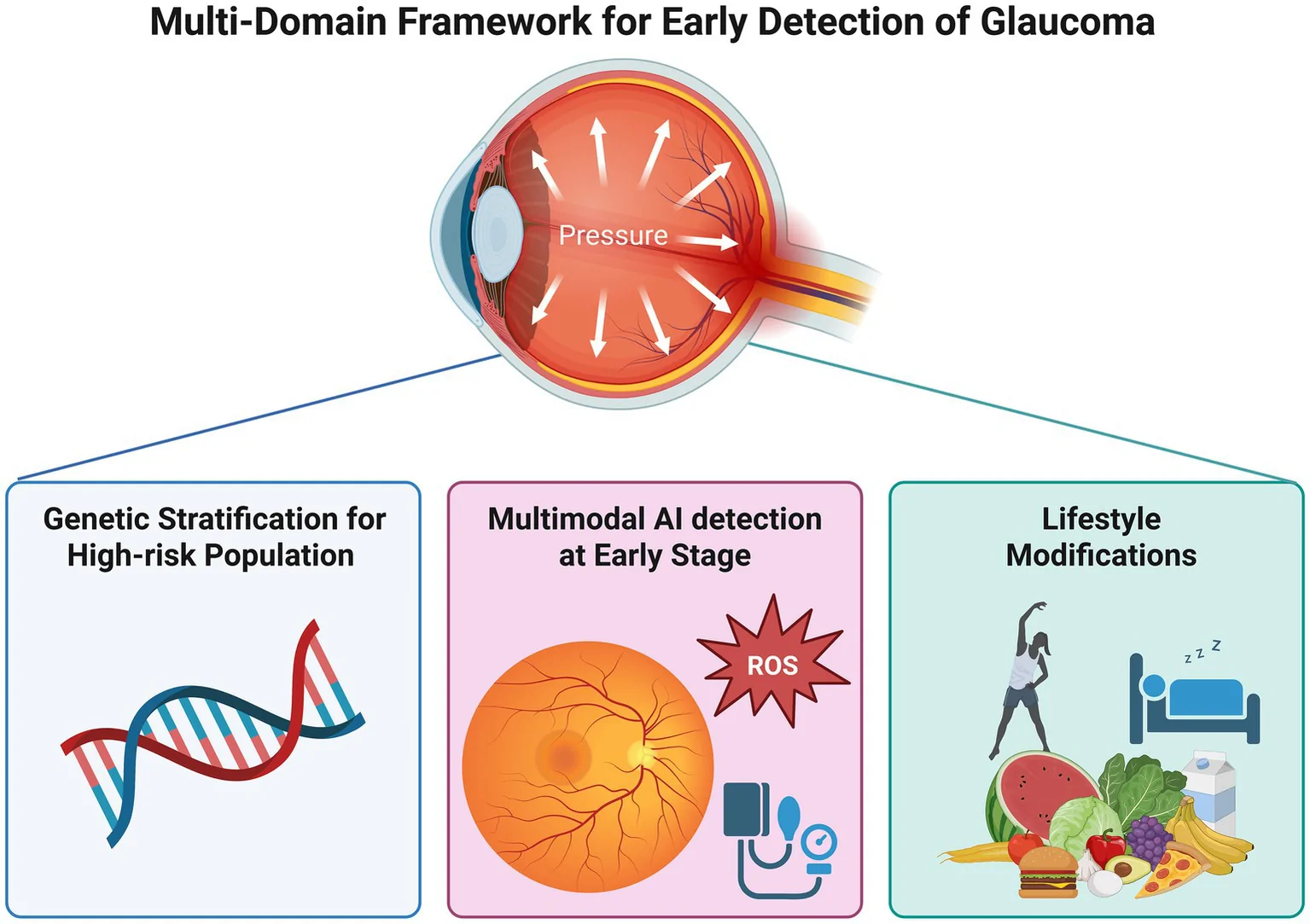
Multidomain framework for early detection of glaucoma. Glaucoma is recognized as a heterogeneous neurodegenerative disorder with multifactorial etiology, involving genetic susceptibility, vascular dysregulation, and modifiable lifestyle factors. Created in BioRender. Hanyuda, A. (2026). https://BioRender.com/ypb4480.

This article synthesizes epidemiological evidence bearing on the case for reorienting glaucoma case detection toward retinal imaging embedded in routine annual health checkups, complemented by multidomain characterization of high-risk individuals. Neither element, however, can be delivered by the clinic-based model that currently defines ophthalmic care. We therefore introduce the *Living Lab*, a community science platform developed under the Japan Science and Technology Agency (JST) COI-NEXT ‘Vision to Connect’ initiative organized by Tohoku University (5), as the setting in which both become feasible (Figure 1).

## 2 The epidemiological imperative: why current screening strategies fall short

### 2.1 The diagnostic gap in Glaucoma

The prevalence of glaucoma in Japan is estimated at approximately 5% among individuals aged 40 years and older, rising steeply with age (3). The Tajimi Study, a population-representative cross-sectional study in central Japan, reported an overall POAG prevalence of 3.9%, of which NTG accounted for approximately 72% of all POAG cases (3). The Hisayama Study, a more recent population-based survey conducted in a community adjacent to Fukuoka, reported an even higher overall glaucoma prevalence of 7.6%, with POAG accounting for 5.8% of adults aged 40 years and older (4). According to both studies, the proportion of undetected cases was alarmingly high, exceeding 80%.

These findings indicate that in a community of 1 million adults aged over 40, tens of thousands of individuals are silently losing their vision without awareness. Given that lost retinal ganglion cells and their axons do not regenerate, every year of delayed diagnosis represents irretrievable structural damage (2).

### 2.2 Inadequate screening for NTG

The canonical risk factor for glaucoma development and progression is elevated IOP. However, the conceptual and practical limitations of IOP-centric screening are particularly acute in the context of NTG. By definition, individuals with NTG have IOP measurements within the statistically normal range (typically defined as ≤21 mmHg) (6). Consequently, they are systematically excluded from identification by screening programs that rely on IOP thresholds.

While IOP lowering remains the only proven treatment modality and its efficacy in NTG has been established by the Collaborative Normal-Tension Glaucoma Study, this therapeutic primacy does not validate IOP as a universal screening parameter (7). A proportion of NTG patients may be involved with additional mechanisms, including vascular dysregulation (8), compromised ocular perfusion pressure (9), systemic hemodynamic instability (10), and neuroinflammation (11). Screening strategies must therefore look beyond IOP if they are to capture this disease burden.

Image-based assessment of the optic disc, whether by fundus photography or optical coherence tomography (OCT), offers a fundamentally different and more inclusive approach to case detection. Optic disc changes, particularly vertical cup-to-disc ratio enlargement, rim notching, and retinal nerve fiber layer (RNFL) thinning, are observable even in NTG patients with entirely normal IOP. The challenge lies in integrating retinal imaging into health-check infrastructure at scale.

## 3 Retinal imaging in annual health checkups: an underutilized opportunity

Japan has one of the most extensively developed annual health checkup (‘*tokutei kenshin*’) systems in the world, mandated by law for employees and covered individuals within the social insurance framework (12). Yet despite this infrastructure, fundus photography for glaucoma detection is not uniformly included in standard checkup protocols.

Incorporating non-mydriatic fundus photography, with automated analysis or telemedicine-based expert reading, into annual checkup programs represents a pragmatic candidate strategy for narrowing the diagnostic gap, the effectiveness and cost-effectiveness of which remain to be established. Evidence from screening programs using non-mydriatic retinal cameras supports the feasibility of this approach in community and occupational health settings (13). Advances in AI-assisted fundus image analysis now offer the prospect of scalable, consistent grading that does not depend on the availability of specialized ophthalmic expertise at point of measurement (14, 15).

The economic case is not yet settled. Direct costs of glaucoma care rise steeply with disease severity (16), but this does not establish that screening is cost-effective, and formal evaluations differ: population screening was not cost-effective in the United Kingdom assessment, although targeted screening of higher-risk groups might be (17), and in the Japanese checkup setting, ophthalmic screening for age-related macular degeneration exceeded conventional willingness-to-pay thresholds (18) whereas AI-assisted diabetic retinopathy screening did not (19). Cost-effectiveness improves when the imaging encounter already exists and when a single image informs several diseases (20).

We therefore propose that incorporation of non-mydriatic fundus photography into the annual health checkup be prospectively evaluated rather than adopted (21). A Japan-specific cost–utility analysis is a planned output of the COI-NEXT ‘Vision to Connect’ hub (5), whose research and development agenda explicitly targets the establishment of future-oriented health screening approaches built around ocular assessment.

## 4 Characterizing high-risk populations: a multidomain approach

The assessments described in this section apply to different populations, and we distinguish them explicitly. Retinal imaging within the annual health checkup is proposed for adults aged 40 years and over. The multidomain characterization that follows is proposed only for individuals identified as higher risk, whether by that imaging or by established risk factors, and not for the examined population as a whole. Genomic, oxidative, and OCT angiography (OCTA) measures are confined to consenting research participants. These distinctions describe the framework as proposed; how they are currently applied in the *Living Lab* is set out below, and the criteria defining higher risk, including the referral threshold applied at the imaging stage, are provisional and are the subject of the validation work in progress.

### 4.1 Genomic risk

Genome-wide association studies (GWAS) have identified numerous loci associated with glaucoma susceptibility and related endophenotypes, including IOP, optic disc morphology, and RNFL thickness (22). Variants in genes such as *MYOC*, *OPTN*, *TBK1*, *CDKN2B-AS1*, *SIX1/SIX6*, *TMCO1*, and *AFAP1* have been robustly associated with POAG risk across multiple ancestral populations (22). Polygenic risk scores (PRS) derived from GWAS summary statistics show meaningful discriminative ability for glaucoma, and their utility in stratifying individuals for targeted surveillance is an area of active investigation (23, 24).

For NTG specifically, genetic architecture may involve loci related to vascular regulation, connective tissue biology and neurodegeneration (25, 26). Variants in genes encoding proteins implicated in ocular blood flow regulation, including those related to endothelin signaling and nitric oxide metabolism, are of particular interest. Integration of genomic risk information into screening programs may in future offer the potential to identify individuals at elevated lifetime risk who may benefit from earlier or more intensive surveillance.

### 4.2 Oxidative stress biomarkers

Oxidative stress has emerged as an important pathophysiology in glaucomatous neurodegeneration (27). Elevated reactive oxygen species (ROS) and reduced antioxidant capacity have been detected in the aqueous humor (28), trabecular meshwork (29), and serum of glaucoma patients relative to healthy controls (30). The retinal ganglion cells (RGCs), with their high metabolic demand and limited regenerative capacity, are particularly vulnerable to oxidative injury.

Circulating biomarkers of oxidative stress—such as malondialdehyde (MDA), 8-hydroxy-2′-deoxyguanosine (8-OHdG), and oxidized low-density lipoprotein—offer accessible surrogate measures of systemic redox imbalance. Measurement of antioxidant capacity through assays such as total antioxidant status (TAS) or glutathione peroxidase activity provides complementary information (31). These measures remain investigational. No circulating redox marker has been prospectively evaluated for predicting incident glaucoma, and none is validated for routine population screening; their collection in the *Living Lab* is confined to consented research protocols.

### 4.3 Systemic lifestyle factors

Epidemiological evidence supports associations between glaucoma risk and a range of systemic and lifestyle parameters, including systemic hypertension (32), diabetes mellitus (33), migraine (34), sleep apnea syndrome (35), myopia (36), and physical activity levels (37). The relationship between systemic blood pressure and NTG is of particular mechanistic relevance: nocturnal blood pressure dips—a common feature of cardiovascular autonomic dysregulation—may reduce ocular perfusion pressure during sleep and thereby contribute to ischemic optic nerve injury in susceptible individuals (32).

Dietary patterns also merit consideration. Epidemiological data on dietary antioxidants and glaucoma risk remain inconclusive. The large population-based cohort studies from the Nurses’ Health Study and the Health Professionals Follow-up Study, found no significant association between intake of carotenoids or antioxidant vitamins and POAG risk (38). Nonetheless, preclinical evidence consistently supports an oxidative injury mechanism in retinal ganglion cell death, and a protective trend has been suggested in some observational analyses of antioxidant intake (39).

Physical inactivity and sedentary behavior are associated with both elevated IOP and compromised vascular regulation (37). A comprehensive lifestyle assessment encompassing dietary quality, physical activity, sleep patterns, body mass index, and smoking status, thus provides actionable data that can simultaneously inform glaucoma risk stratification and broader preventive health counseling (40).

### 4.4 Ocular blood flow dynamics

Vascular insufficiency to the optic nerve head is increasingly recognized as a central mechanism in NTG pathogenesis. Ocular perfusion pressure (OPP), more precisely defined as two-thirds of mean arterial pressure minus IOP [mean OPP = 2/3 × (diastolic blood pressure + 1/3 × (systolic blood pressure – diastolic blood pressure)) − IOP], reflects the driving force for blood delivery to the retina and optic nerve head (9). Reduced OPP, whether through low systemic blood pressure, IOP fluctuation, or impaired vascular autoregulation, has been associated with greater optic nerve damage in NTG patients (9).

Advanced imaging modalities including OCTA now permit non-invasive quantification of optic disc and macular perfusion with high reproducibility. Reduced radial peripapillary capillary flow density measured by OCTA is an early finding in NTG that may precede detectable RNFL thinning on structural OCT. (41) We do not, however, propose OCTA for risk stratification. Devices are confined to specialist centers, cost an order of magnitude more than a fundus camera, and no community-deployable instrument exists; nor has any study shown added predictive value beyond structural OCT in an unselected population. Its use here is confined to consented research protocols.

## 5 The *living lab*: a model for community science co-creation

### 5.1 Rationale and vision

The epidemiological and clinical arguments outlined above identify a clear need: effective glaucoma prevention requires population-level health data collection, prospective surveillance, multidomain risk characterization, and engagement of individuals who are currently outside the healthcare system. These objectives are not achievable through the conventional clinic-hospital model alone. They require a new kind of infrastructure—one that is embedded in daily community life, accessible without appointment, and built around the needs and motivations of citizens as active participants rather than passive recipients of care.

**Figure 2:**
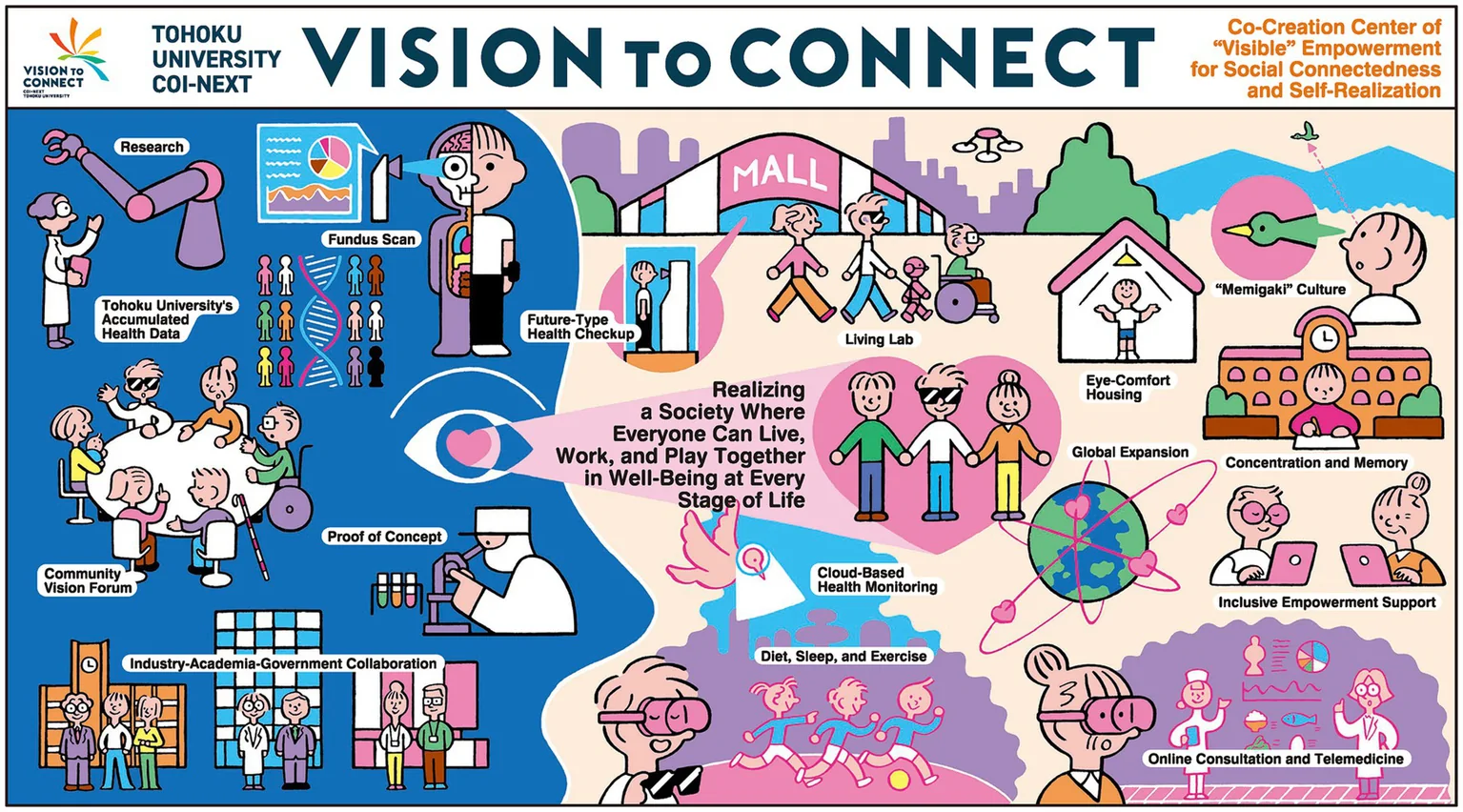
Schematic overview of the COI-NEXT ‘Vision to Connect’. This illustration represents the envisioned future of the hub in 10 to 20 years.

The *Living Lab* (‘neighborhood health lab’) was conceived and established as precisely such an infrastructure. Developed under the COI-NEXT ‘Vision to Connect’ hub at Tohoku University, a program designed to foster co-creation among academia, industry, and society, the *Living Lab* opened its inaugural site in July 2024 within the AEON Tomiya shopping center (Tomiya City, Miyagi Prefecture, Japan), and subsequently expanded to AEON Style Sendai Kamisugi (5) (Figure 2). The choice of a commercial retail environment was deliberate: shopping centers attract broad demographic cross-sections, reduce the psychological and logistical barriers associated with clinical settings, and enable opportunistic health engagement during routine daily activities. The setting also determines which of the risk domains described above can realistically be assessed outside a clinic.

### 5.2 Operational model and measurements

The *Living Lab* operates on two levels of access, aligned with the IOP-independent risk domains described above. Routine, no-appointment access centers on non-medical devices targeting systemic and vascular phenotypes most relevant to NTG including autonomic dysfunction evaluation using the Kiritsunameijin system (42), cutaneous advanced glycation end-products accumulation (43, 44), digital vegetable intake estimation, capillary microvascular observation (45), visual field (46, 47) and practical visual acuity screening, and a mobile self-check application. A weekly physician-supervised session adds medical-grade assessment with fundus OCT (48) and axial length measurement (49), together with real-time telemedicine consultation. The consultation serves a dual purpose: it provides expert interpretation on site, and it lowers the psychological threshold for formal clinic visits among participants who might otherwise defer evaluation. Where indicated, participants are referred to ophthalmology clinics for definitive workup. The *Living Lab* thus functions as a bridge between community-based multidomain risk profiling and specialist care.

Since the inaugural site opened in July 2024, the *Living Lab* has received approximately 600 visitors. Of these, 486 underwent fundus photography and were enrolled in a prospective observational study. Optic disc findings consistent with glaucoma suspicion were identified in 121 participants (24.9% of those imaged), of whom 85% had been unaware of any ocular abnormality before attending. Following advice given during the on-site health consultation, 56.3% of these participants subsequently attended an ophthalmology clinic for definitive evaluation. These figures are descriptive and derive from self-selected shopping-center visitors without a defined denominator population. Further validation is currently ongoing.

As currently implemented, this model does not apply the risk-based distinctions set out above. The non-medical assessments are available to all attending participants without appointment, while OCT/OCTA and axial length measurements are obtained during the weekly physician-supervised session; the division therefore reflects the availability of medical supervision rather than assessed risk. This is deliberate at the present exploratory stage, since determining which measures contribute to risk discrimination requires that they be obtained across the attending population rather than only within a preselected subgroup. Establishing the criteria by which the proposed distinctions should later be applied is an objective of the validation work in progress.

### 5.3 A multi-stakeholder value proposition

A defining feature of the *Living Lab* model is the explicit alignment of value creation across four distinct stakeholder groups, each of whose participation is mutually reinforcing.

For citizens, the laboratory addresses a fundamental anxiety about health status that often goes unmet outside formal clinical encounters. Community members gain access to ophthalmological and general health measurements they would not routinely receive, with immediate feedback and, where relevant, medical guidance. Participation in longitudinal health observation research simultaneously confers the psychosocial value of social contribution and scientific engagement—a form of participatory citizenship in health.

For industry partners, including health food, nutraceutical, ophthalmic device, and pharmaceutical companies, the *Living Lab* offers a platform for evidence-based health product development anchored in real-world longitudinal data. The *Ronbun Recipe®* concept, developed collaboratively between Tohoku University and AEON Tohoku, exemplifies this: food products incorporating ingredients with documented health-promoting properties in published scientific literature are prepared and sold to consumers, creating a direct pathway from evidence to application and embodying the vision of ‘science you can eat’ (50). Following collaborative development and trademark registration, proof-of-concept initiatives have been implemented across AEON supermarkets, local café buffets, and a Rohto Pharmaceutical-operated restaurant. The collaboration equips chefs with the underlying scientific evidence, with the shared goal of shifting consumer perception from food that merely appears healthy toward cuisine that is both appetizing and genuinely beneficial to health outcomes.

For researchers, the *Living Lab* constitutes a novel longitudinal cohort infrastructure with a distinctive advantage over academic and clinical settings: it enables recruitment of healthy individuals and asymptomatic, undiagnosed persons who rarely enter conventional research pipelines anchored to hospital visits. The prospective collection of multimodal health data, including ophthalmological, physiological, biochemical, genomic, and lifestyle measures, in a community-dwelling adult population provides the substrate for high-impact epidemiological and translational research. The open-ended nature of the cohort accommodates emerging research questions, and the embedded measurement infrastructure reduces the logistical barriers to data collection.

For local municipalities and public health authorities, the *Living Lab* directly addresses the policy imperative of healthcare expenditure rationalization. Early detection of glaucoma, together with the systemic conditions that co-occur with it, may reduce downstream treatment costs and disability-associated expenditures. By converting a commercial space into a health monitoring hub, the model also advances health equity goals, reaching individuals who do not typically engage with preventive health services.

## 6 Discussion: toward an integrated preventive framework

The convergence of arguments presented in this article points toward a coherent preventive framework for glaucoma in the Japanese context, in which two established propositions are joined by the element that makes them deliverable. The first is systematic inclusion of retinal imaging in annual health checkups as the primary means of population-level case detection; the second is multidomain characterization of individuals at higher risk, integrating genomic, oxidative, lifestyle and hemodynamic dimensions. Neither can be delivered by the clinic-based model that currently defines ophthalmic care, and it is on this point that the framework depends: community-embedded infrastructure, exemplified by the *Living Lab*, is what allows both to reach populations that do not routinely present for ophthalmological assessment — and it is conceived not as a screening station but as a platform for well-being, within which glaucoma is one condition detected.

This framework departs from the prevailing clinic-centric model in several important respects. It reconceptualizes the locus of prevention from specialist outpatient departments to community retail and commercial settings. It reframes citizen participation from passive receipt of screening results to active engagement in a shared health knowledge enterprise. And it repositions industry as a partner in evidence generation rather than solely as a purveyor of products to a defined patient market.

Established programs address parts of the same problem. Singapore’s Integrated Diabetic Retinopathy Programme applies glaucoma-suspect referral criteria to photographs acquired for retinopathy screening (51), and the pyramidal primary eye-care networks developed in India link community vision centers to tertiary hospitals by teleconsultation (52). Both differ from the present model in whom they reach: the former recruits from a disease register, and the latter addresses a shortage of geographical and economic access. In Japan access is not the binding constraint — what is missing is any reason for an asymptomatic person to attend — and the *Living Lab* is designed against non-attendance rather than unavailability. What these programs possess and the *Living Lab* does not is a defined denominator population, published quality-assurance standards, and routine reporting of referral outcomes; adopting these is the next stage of development.

Several implementation challenges require acknowledgment. The quality assurance of fundus photography in non-clinical settings demands robust training protocols for operators and validated automated or telemedicine-based image grading pipelines. The translation of genomic risk information into actionable clinical guidance requires careful attention to health literacy, cultural context, and the prevention of stigmatization or unwarranted alarm. Privacy protections for longitudinal health data collected in commercial settings must meet rigorous standards. And the sustained engagement of community participants in a longitudinal cohort over years to decades presents motivational and logistical challenges that require innovative solutions including, potentially, the gamification and immediate health feedback mechanisms already incorporated into the *Living Lab*.

These constraints take different forms elsewhere. Embedding retinal imaging in a statutory annual checkup is directly applicable in the Republic of Korea and Taiwan, which operate comparable national examination programs, but not in systems lacking such infrastructure. The community platform is far less dependent on existing infrastructure and is therefore the more portable element. In low- and middle-income settings it would nonetheless require substantial reduction: the genomic, oxidative and OCTA components are research instruments and should be omitted, leaving non-mydriatic imaging by a trained non-specialist, automated or telemedicine-based grading, and a referral pathway. The last is the binding constraint, since detection without accessible treatment capacity confers no benefit and may cause harm; the prerequisite is therefore capacity for glaucoma care, not imaging hardware.

In Japan, the constraint is neither imaging capacity nor treatment capacity, but the absence of any occasion on which an asymptomatic person is examined (3). Japan’s universal health insurance system faces mounting strain as the population ages (53), and irreversible neurodegenerative conditions of later life, such as glaucoma and dementia, demand a shift from reactive treatment toward early detection and high-risk-targeted prevention. The documented inadequacy of current glaucoma detection rates (3), combined with the availability of increasingly affordable imaging and biosensor technologies, makes this a reasonable direction to test. What is ultimately required, however, is a civic culture in which citizens take the initiative in their own preventive care. Whether a community platform can sustain the voluntary participation on which such a culture depends is the question the *Living Lab* was built to answer, and it is not yet answered.

## 7 Conclusion

Glaucoma, particularly NTG, imposes a substantial and largely undetected burden of visual disability in Japan. The limitations of IOP-based screening, combined with the asymptomatic progression of early disease, have created a diagnostic gap that will not be closed by incremental adjustments to the current clinic-based model. A meaningful response may require systematic retinal imaging in annual health checkups, multidimensional characterization of high-risk populations, and novel community-based infrastructure for longitudinal health monitoring.

The *Living Lab* represents an early attempt to test the feasibility of a new paradigm in which prevention science is not confined to the hospital or laboratory but is woven into the fabric of daily community life. The *Ronbun Recipe®* initiative further illustrates how evidence-based health knowledge can be translated into accessible, enjoyable consumer experiences, closing the loop between scientific discovery and public health benefit. We believe that a model in which communities become active participants in health science, rather than passive recipients of medical care, represents a promising solution to the compounding challenges of population aging and declining birth rates in Japan.

## Data Availability

The original contributions presented in the study are included in the article/supplementary material, further inquiries can be directed to the corresponding author.
